# Potential Activity of 3-(2-Chlorophenyl)-1-phenyl-propenonein Accelerating Wound Healing in Rats

**DOI:** 10.1155/2014/792086

**Published:** 2014-01-22

**Authors:** Summaya M. Dhiyaaldeen, Mohammed A. Alshawsh, Suzy M. Salama, Nahla S. I. Alwajeeh, Rami Al Batran, Salmah Ismail, Mahmood Ameen Abdulla

**Affiliations:** ^1^Department of Biomedical Science, Faculty of Medicine, University of Malaya, 50603 Kuala Lumpur, Malaysia; ^2^Department of Pharmacology, Faculty of Medicine, University of Malaya, 50603 Kuala Lumpur, Malaysia; ^3^Center of Studies for Periodontology, Faculty of Dentistry, University Technology MARA (UiTM), 40450 Shah Alam, Selangor, Malaysia; ^4^Institute of Biological Sciences, Faculty of Science, University of Malaya, 50603 Kuala Lumpur, Malaysia

## Abstract

Wound healing involves inflammation followed by granular tissue development and scar formation. In this study, synthetic chalcone 3-(2-Chlorophenyl)-1-phenyl-propenone (CPPP) was investigated for a potential role in enhancing wound healing and closure. Twenty-four male rats were divided randomly into 4 groups: carboxymethyl cellulose (CMC) (0.2 mL), Intrasite gel, and CPPP (25 or 50 mg/mL). Gross morphology, wounds treatment with the CPPP, and Intrasite gel accelerate the rate of wound healing compared to CMC group. Ten days after surgery, the animals were sacrificed. Histological assessment revealed that the wounds treated with CPPP showed that wound closure site contained little amount of scar and the granulation tissue contained more collagen and less inflammatory cells than wound treated with CMC. This finding was confirmed with Masson's trichrome staining. The antioxidant defence enzymes catalase (CAT) and superoxide dismutase (SOD) were significantly increased in the wound homogenates treated with CPPP (*P* < 0.05) compared to CMC treated group. However, in the CPPP treatment group, lipid peroxidation (MDA) was significantly decreased (*P* < 0.05), suggesting that the CPPP also has an important role in protection against lipid peroxidation-induced skin injury after ten days of treatment with CPPP, which is similar to the values of cytokines TGF-**β** and TNF-**α** in tissue homogenate. Finally the administration of CPPP at a dosage of 25 and 50 mg/kg was suitable for the stimulation of wound healing.

## 1. Introduction

The process of wound healing and repair is a response to the dermal skin injury. Once damaged, the inflammatory cells response starts, and the fibroblast cells adjacent to injury start to proliferate and synthesize collagen fibers and epithelization [1]. It is well known that immune-mediated physiologic mechanism played a significant role in wound healing and repair [2]. Wound-healing and repair is auto-process in which skin heals itself after damage [3]. The extracellular matrix (ECM) in wound area is a complex structure that supports cells and is a key component of the basement membrane, which helps to anchor and replenish epidermal cells in healthy skin. During the wound-healing process, the ECM governs biological responses throughout the 4 healing phases: haemostasis, inflammation, proliferation, and remodelling. The effects of the various ECM components vary in different wound stages and are influenced by cell signalling and growth factors in a dynamic, reciprocal process [2]. In folk medicine, several herbs and medicinal plants have been used traditionally to treat variety of skin infection and injuries externally, including wounds [4–8].

**Figure 1:**
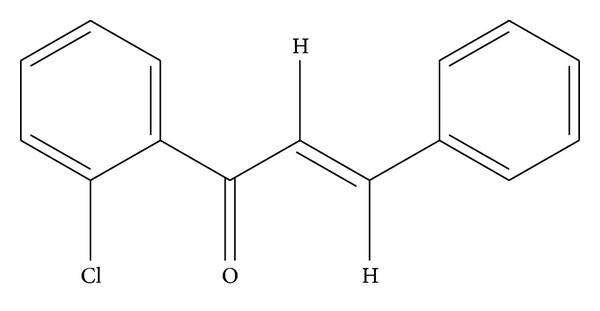
Chemical structure of CPPP [3-(2-chlorophenyl)-1-phenyl-propenone].

Chalcones act as a precursor in the synthesis and characterization of a large number of biologically important heterocycles such as pyrazolines, benzothiazepine, 1,4-diketones, and flavones. Synthesis and characterization of chalcones provided significant advantages to organic and medicinal chemists [9]. The chemical structure of CPPP is shown in Figure 1. Chalcones are *α*, *β*-unsaturated ketones composed of 2 aromatic rings (A and B) having diverse array of substituents. Chalcones possess significantly a broad spectrum of pharmacological and biological activities, among which anti-infective, anti-inflammatory [10], antimalarial [11], and antioxidant activities have been reported [12]. They have shown antimitotic and antiproliferative activities also [13]. The aim of the present study was to assess the influence of CPPP in rate of wound healing enclosure in rodents.

## 2. Materials and Methods

### 2.1. Materials

Intrasite gel (a trademark for Smith and Nephew Healthcare Limited) was purchased from the Experimental Animal House Unit, Faculty of Medicine, University of Malaya. Catalase, SOD, and MDA commercial kits were purchased from the Cayman Chemical Company (Cayman, USA). 3-(2-Chlorophenyl)-1-phenyl-propenone (CPPP) chalcone was obtained from Pharmacology Department, Faculty of Medicine, UM. To prepare 2% CMC, 2 gram of CMC was dissolved in 100 mL of sterile distilled water. Then, the CPPP chalcone was dissolved in 2% CMC and oral gavages at a dosage of 25 and 50 (mg/kg) body weights according to the recommendation of Shetty et al. [14].

### 2.2. Acute Toxicity

Healthy male and female SD rats (2 months old) were purchased from the Experimental Animal House Unit (University of Malaya, Ethics no. PM/29/06/2012/SMD (R)). The animals weighed between 150 and 180 g. The rats were fed standard pellets and allowed access to tap water and placed individually in separate cages with wire bottom. A total of 48 SD rats (24 males and 24 females) were equally divided into 4 groups and oral gavages, respectively, with 0.5% CMC vehicle, 250, 500, and 1000 mg/kg of CPPP. The animals fasted for 16 hours prior to oral dosing. Food was withheld for a further 3 to 4 h after oral gavage. All SD rats were watched for 24 and 48 hours after the oral route of the CPPP solution for behavioral changes and toxicological symptoms. Mortality, if any, was recorded and the rats were sacrificed by an overdose of ketamine and xylazine anesthesia upon completion of the 15th day. Histology and biochemical parameters of liver and kidney were determined as described in [15]. Throughout the studies, all experimental rats were treated humanely according to the criteria outlined in the “Guide for the Care and Use of Laboratory Animals” [16].

### 2.3. Laboratory Animals

Adult male SD rats were purchased from the Experimental Animal House Unit. Four groups of SD rats were randomly divided, six animals per group. Each rat weighed between 250 and 280 g (8 weeks old) and was housed separately in cage with wire mesh bottom, with 1 rat per cage. The animals were fed standard pellet and allowed access to tap water. This study was approved by the Ethics Committee for Animal Experiment (Ethic no. PM/29/06/2012/SMD (R)).

### 2.4. Experimentally Induced Wounds

The rats were anaesthetised by intramuscular injection of ketamine and xylazine prior to creating wounds. The skin was clipped and disinfected with 70% alcohol. Two cm of skin uniform in diameter was removed from the area of dorsal neck with the aid of a round seal under general anaesthesia. Incisions in the tissues underneath the wound area were avoided and the skin tension was kept constant during the experiment. The entire wound was left open, and the wound area at day 0 was measured immediately after wound creation by the aid of transparent tracing paper. The transparent paper was then placed on a 1 mm^2^-grid graph sheet and was recorded as recommended previously [17].

### 2.5. Topical Application of Vehicles

Animals in Group 1 were topically treated with 0.2 mL of the vehicle, 2% w/v CMC, twice daily as a placebo control group [18]. Group 2 rats were dressed twice daily with 0.2 mL of Intrasite gel as a positive control. Animals of Groups 3 and 4 were dressed twice daily with 0.2 mL of the 25 or 50 mg/kg CPPP in vehicle, respectively. The wounds were observed daily till the end of the experiment.

### 2.6. Estimation of the Rate of Wound Closure

The wound closure areas were assessed manually via transparent tracing paper on days 0, 5, and 10 were expressed as the percentage of wound healing as suggested previously [17]. The wound areas were measured using graph paper. The percentage of wound closure area was determined for each day. The duration required for forming a scar at wound closure area without any residual raw wound was termed the epithelisation period.

### 2.7. Preparation of Tissue Homogenate

Wound tissues were washed with ice-cold normal saline. With the aid of a homogenizer (Polytron, Heidolph RZR 1, Germany), tissue homogenate was prepared by adding mammalian protease blockers. The tissue homogenate was spine for 30 minutes at 4°C.

### 2.8. Antioxidant Measurements from Granulation Tissues

#### 2.8.1. Assessment of SOD

The SOD activity was assessed via Cayman kit (Cayman, USA) according to the guideline procedures [19].

#### 2.8.2. Measurement of Catalase (CAT)

Catalase activity was evaluated using Cayman kit (Cayman, USA) according to the manufacturer guideline.

### 2.9. Lipid Peroxidation from Granulation Tissues

Tissue Malondialdehyde (MDA) (nmol/mL) was determined using Cayman kit (Cayman, USA) according to the manufacturer guideline [20].

### 2.10. Enzyme-Linked Immunosorbent Assay (ELISA)

Wound tissue homogenate was used for assessment of TNF-*α* and TGF-*β*, according to the manufacturers' instructions (Abnova, USA).

### 2.11. Histopathological Evaluation of Wound Area

The wound tissues with normal skin adjacent to wound site were fixed in 10% of phosphate buffered formalin and processed for routine histological method and stained with a general routine stains (haematoxylin and eosin) and examined under light microscope.

### 2.12. Masson's Trichrome Staining

This staining method (Sigma, USA) stained the deposition of collagen fibres with greenish blue colour in granulation tissues just below the wound area. Images analysis has been done by two experienced observers in an independent and blinded fashion for the control and the experimental groups.

### 2.13. Statistical Analysis

All values were represented as means ± SEM. Values were analysed using analysis of variance (ANOVA) then by Tukey's post hoc for multiple comparisons. Values are significant at *P* < 0.05.

## 3. Results

### 3.1. Evaluation of Acute Toxicity

No significant toxicity or death existed between groups throughout the experiment. Histology, liver, and kidney showed no hepatotoxicity or nephrotoxicity between groups. Biochemical parameters were within the normal ranges and no differences between groups.

### 3.2. Evaluation of Wound Healing

**Figure 2:**
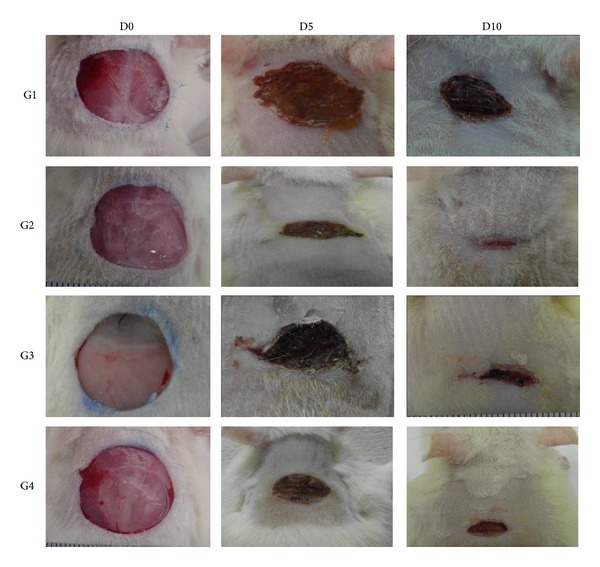
Effect of CPPP on the gross appearance of wound healing on day 0, 5, and 10. (G1) The CMC group, showing incomplete wound healing. (G2) The Intrasite gel group, showing complete wound healing. (G3) The 25 mg/kg CPPP group, showing complete wound healing. (G4) The 50 mg/kg CPPP group, showing complete wound healing.

Grossly, the wounds dressed with Intrasite gel (Group 2) revealed remarkable wound repair and the rate of healing significantly accelerated compared to that of control group (Group 1). Group 2 had the highest rate of healing among all groups. Wounds dressed with 50 mg/kg of CPPP achieved a wound-healing rate equivalent to the healing rate of Group 2. Rats treated with 25 mg/kg CPPP had a faster wound-healing rate than rats in Group 1 but a slower wound-healing rate than rats in Groups 2 and 4 (Figure 2). These findings suggest that a high dose of CPPP may be as effective as Intrasite gel in improving wound-healing progression.

Wound closure was measured to determine the percentage of wound healing in each rat (Table 1). During the study, the wound closure percentage in the CMC-treated group was significantly less compared to CPPP or Intrasite gel treatment. The rats given the high dose of CPPP (Group 4) had a comparable level of healing to the rats given the Intrasite gel (Group 2) and a greater level of healing than the rats given the low dose of CPPP (Group 3). These evaluations provide further independent confirmation that CPPP treatment effectively improves skin wounds in a dose-dependent manner.

### 3.3. Antioxidant Enzymes Activities and Level of MDA in Wound Tissue Homogenate

**Figure 3:**
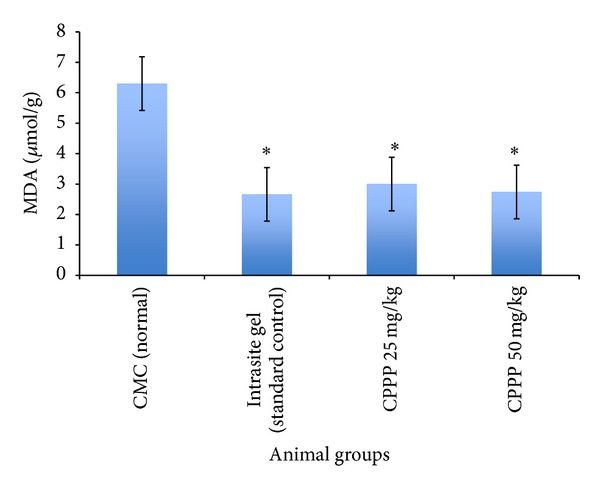
Effects of CPPP on the content of MDA in tissue homogenates of dermal rat wounds. Statistical analysis of the data was performed using one-way analysis of variance (ANOVA) and Dennett's post hoc test for average comparisons on SPSS 18.0. Mean values ± SEM were used. Significance was defined as *P < 0.05 compared to G1 (CMC).

The lipid peroxidation parameter (MDA) is shown in Figure 3. Generally, the rats dressed with CMC showed significant (*P* < 0.05) increased levels of lipid peroxidation level compared to the treatment groups. Notably, the animals dressed topically with 50 mg/kg CPPP (Groups 4) showed significantly decreased levels of MDA in wound homogenate compared to the animals treated with the CMC (Group 1). These investigations indicate that wounds dressed with CPPP may protect the skin from lipid peroxidation during the experiment.

The CAT and SOD results were higher in rats dressed with CPPP and Intrasite gel compared to CMC group (Table 2). Rats dressed with Intrasite gel had the highest CAT and SOD levels. Dressing the animals with high dose of CPPP significantly (*P* < 0.05) increased CAT and SOD levels. These results collectively supported the suggestion that CPPP may provide a favourable host environment for accelerating wound healing.

### 3.4. Assessment of Cytokines in Tissue Homogenate

The level of the cytokines TNF-*α* and TGF-*β* from tissue homogenate are shown in Table 3. TGF-*β* levels were reduced significantly in tissue homogenate samples from the CMC group compared with treated groups. Topical application of CPPP to rats increased the fibrogenic factor TGF-*β* levels in both groups of CPPP-treated rats. In contrast, the levels of the pre-inflammatory TNF-*α* reduced in the CPPP-dressed groups comparing to CMC group. Levels of TNF-*α* and TGF-*β* from the 50 mg/kg of CPPP dressed group were comparable to the values of the Intrasite gel-dressed group.

### 3.5. Histopathological Changes

**Figure 4:**
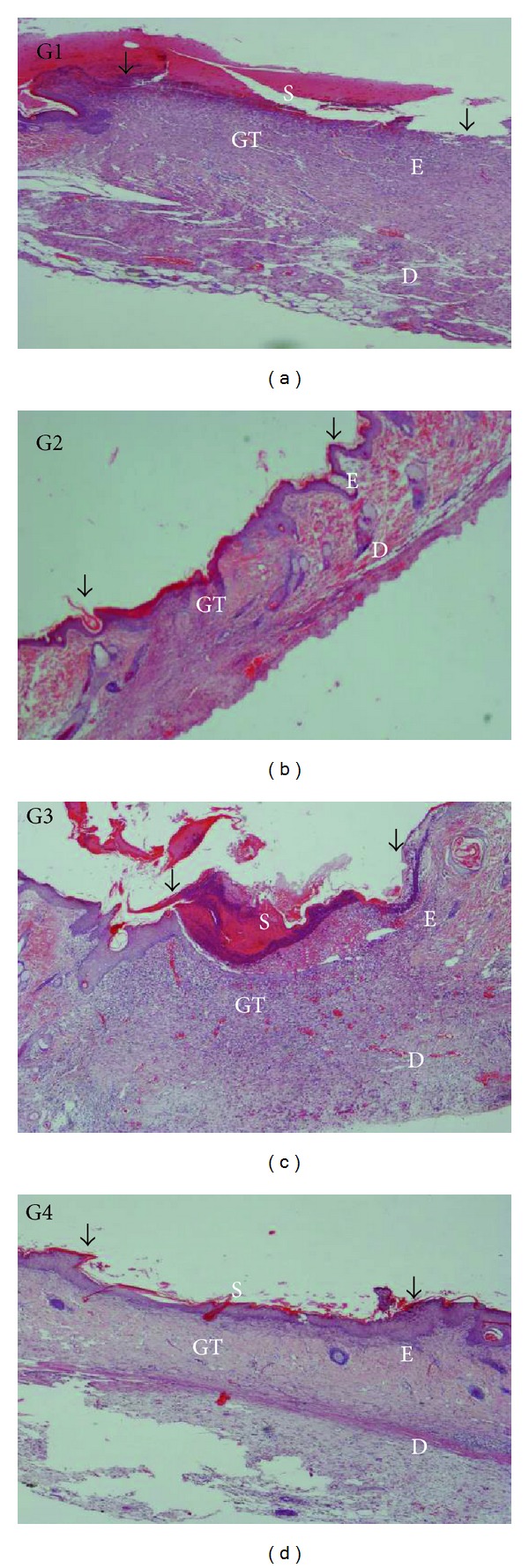
A histological section of a healed wound on day 10 after surgery (H&E stain, 4x magnification). The arrow indicates re-epithelialization. S—Scab; E—Epidermis; D—Dermis; GT—Granulation tissue. (G1) The CMC group, showing incomplete wound healing and closure. (G2) The Intrasite gel group, showing complete wound healing and closure. (G3) The 25 mg/kg CPPP group, showing a narrow scar region and wound closure. (G4) The 50 mg/kg CPPP group, showing complete wound healing and closure.

**Figure 5:**
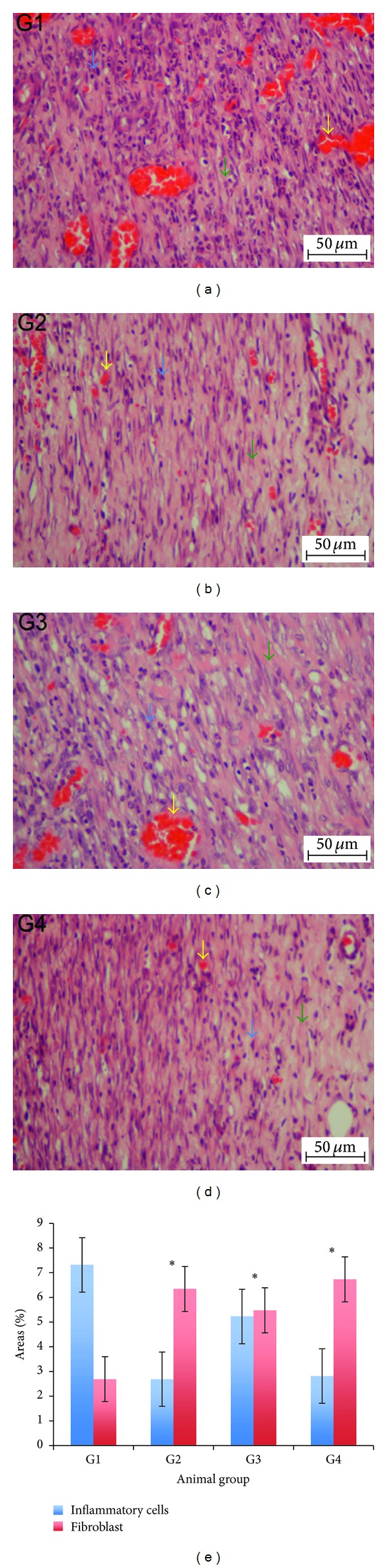
A histological section of a healed wound on day 10 after surgery (H&E stain, 40x magnification). Inflammatory cells (blue arrows); fibroblasts (green arrows); blood vessels (yellow arrows). (G1) The CMC group granulation tissue contains comparatively fewer fibroblasts and more inflammatory cells. (G2) The Intrasite gel group granulation tissue contains comparatively more fibroblasts and few inflammatory cells. (G3) The 25 mg/kg CPPP group granulation tissue has comparatively moderate numbers of fibroblasts and few inflammatory cells. (G4) The 50 mg/kg CPPP group granulation tissue contains comparatively more fibroblasts and few inflammatory cells. Image analysis was executed using an optical image analyzer (Image Pro plus 4.5, Media Cybernetics, Silver Spring, MD). Data was expressed as the mean ± SEM and analyzed using One Way ANOVA followed by Tukey's post hoc test for average comparison on SPSS 18.0. Significance was defined as *P < 0.05 compared to CMC group.

The microscopic assessment of the H&E-stained sections at magnifications of 4x and 40x are shown in Figures 4 and 5, respectively. The microscopic appearance of the skin of the CMC-treated animals (Group 1) revealed greater scar width at 4x magnification. At 40x magnification, these rats had high amounts of inflammatory cell infiltrate and few fibroblasts, blood vessel formation, and collagen deposition.

Skin from the Intrasite gel and the high-dose CPPP-dressed animals (Groups 2 and 4) had smaller scar width at 4x magnification and less inflammatory infiltrate and had more fibroblasts, blood vessel formation, and collagen deposition at 40x magnification.

In contrast, the skin of the 25 mg/kg dosage of CPPP (Group 3) had a smaller scar width at 4x magnification and fewer inflammatory cells and more fibroblasts, blood vessels, and collagen at 40x magnification than the skin of the CMC-treated rats (Group 1). Morphometric image analysis confirmed that wound healing in rats could be effectively improved by CPPP treatment.

### 3.6. Masson's Trichrome Staining

**Figure 6:**
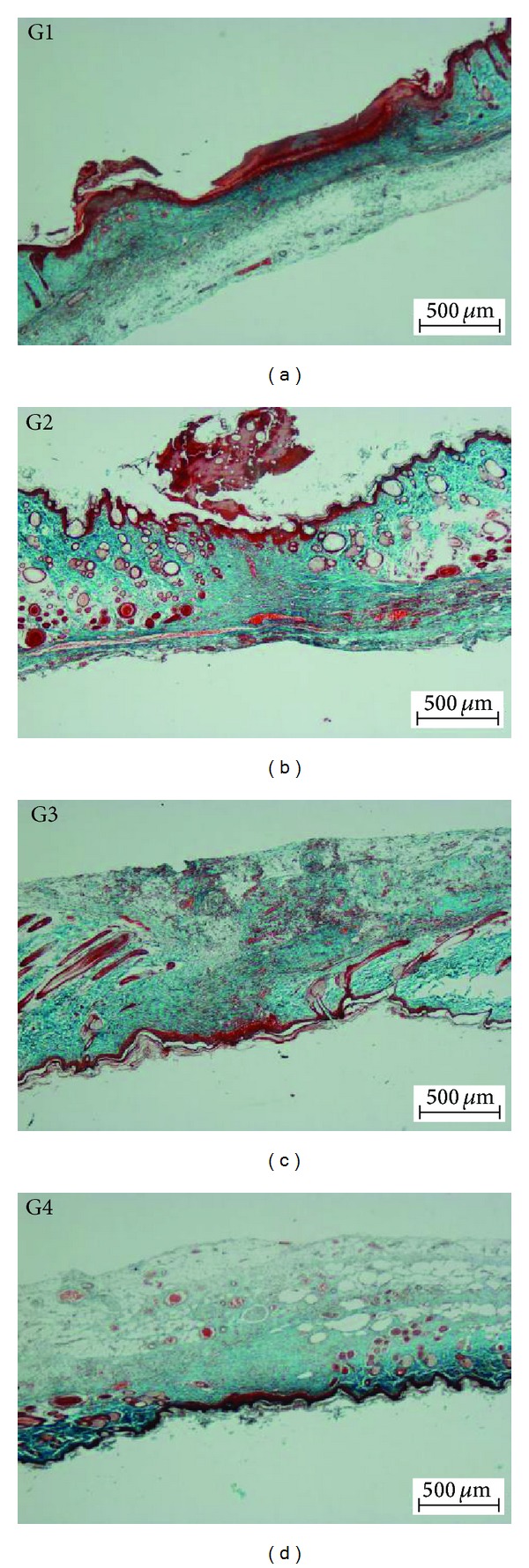
Effect of the CPPP on Masson's trichrome staining of healed wounds on day 10 after surgery. (G1) The CMC group, showing incomplete wound healing and closure. (G2) The Intrasite gel group, showing complete wound healing and closure. (G3) The 25 mg/kg CPPP group, showing a narrow scar region and wound closure. (G4) The 50 mg/kg CPPP group, showing complete wound healing and closure (Masson's trichrome stain, 4x magnification).

**Figure 7:**
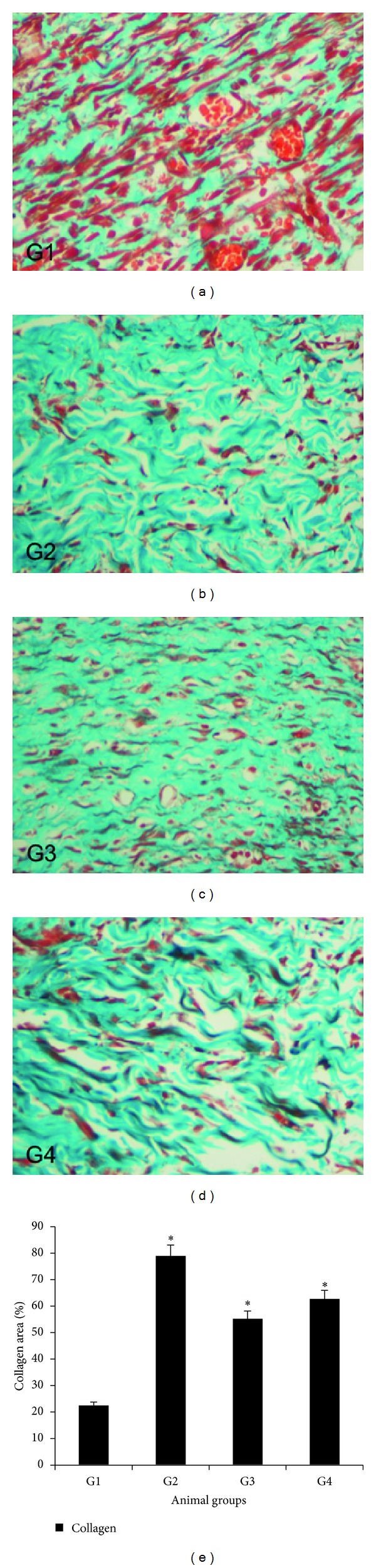
Effect of the CPPP on Masson's trichrome staining of granulation tissue in healed skin wounds on day 10 after surgery. (G1) The CMC group, showing low degrees of collagen deposition and alignment. (G2) The Intrasite gel group, showing greater degrees of collagen deposition and alignment. (G3) The 25 mg/kg CPPP group, showing moderate degrees of collagen deposition and alignment. (G4) The 50 mg/kg CPPP group, showing greater degrees of collagen deposition and alignment (Masson's trichrome stain, 40x magnifications). Image analysis was executed using an optical image analyzer (Image Pro plus 4.5, Media Cybernetics, Silver Spring, MD). Data was expressed as the mean ± SEM and analyzed using One Way ANOVA followed by Tukey's post hoc test for average comparison on SPSS 18.0. Significance was defined as *P < 0.05 compared to CMC group.

The degree of collagen deposition as determined by Masson's trichrome staining is illustrated at magnifications of 4x and 40x in Figures 6 and 7, respectively. Skin tissue sections from the CMC-treated rats appeared to be normal, with minimal signs of collagen deposition. Skin tissues from the Intrasite gel-dressed group showed mild collagen accumulation, indicating that some level of healing was beginning to occur. Skin dressed with 25 mg/kg dosage of CPPP showed moderate collagen accumulation, while skin of high-dose CPPP treated rats showed mild collagen accumulation.

## 4. Discussion

Sprague-Dawley rats were commonly used for wound healing model to evaluate the rate of wound healing and histology of wound areas using different synthesized pure compounds or plant extract and their isolates because rats are easy to handle and more economic than other laboratory animals [21].

Throughout the experiment, the topical application of CPPP did not show any signs of behavioural changes and did not scratch/bite the wound site when CPPP was applied. The process of wound healing is complex process that restores damaged tissue to a normal state and begins in the proliferation of fibroblast and deposition of collagen fibres and angiogenesis in granulation tissue, scar formation, wound contraction, and epithelialisation [22].

Intrasite gel contains 2.3% carboxymethyl cellulose and 20% propylene glycol which act as a humectant and preservative, respectively. Intrasite gel is used for the treatment and management of different types of wounds and skin disorders [23].

Our results demonstrate that the CPPP as a topical applicant to wound areas significantly accelerates the rate of wound healing closure. Histological analysis shows that the granulation tissue of wounds treated with CPPP contains comparatively more collagen, fewer inflammatory cells, and a higher level of angiogenesis. These observations are in agreement with the results published by Zahra et al. [6], who showed that wounds treated with plant extract contain more collagen deposition and fewer inflammatory cells and angiogenesis. Acceleration of wound-healing potential of CPPP may be due to the deposition of more collagen fibres with angiogenesis and less inflammatory cells in granulation tissue of wound area. Similarly, increase in the rate of healing activity has been attributed to angiogenesis and collagen deposition in granulation tissue [18]. Collagen plays a significant role in the process of wound healing activity and is a major component of granulation tissues which enhance tissue regeneration [24]. Increased angiogenesis in granulation tissues just below the wound closure enhanced circulation of blood and brought more oxygen and nutrients required for healing and reepithelisation [25]. Thus, the angiogenesis in granulation tissue enhanced the proliferation epithelial cell which plays a major role in the increase in the rate of wound-healing process [26]. Habibipour et al. [27] showed that treated wounds are characterised by significant collagen synthesis, fibroblast proliferation, and neovascularisation, which increase the tensile strength and accelerate the healing of such wounds. The acceleration of wound-healing potential of CPPP may be due to its flavonoids properties and mainly due to their antimicrobial activity, wound contraction, and increased reepithelialisation rate [28]. Flavonoids decreased lipid peroxidation, prevented cell death, enhanced DNA synthesis, and improved angiogenesis [29].

Various mechanisms of wound healing promote the production of antioxidants enzymes in the wound area and provide a suitable environmental condition for wound healing process [30]. CPPP has been shown to have antioxidant activity [31]. It has been reported that antioxidants play a major role in accelerating the rate of wound healing closure and may play a significant contributory factor for favourable wound healing [27]. Indeed, antioxidants are believed to play a major role in process of wound healing activity and to protect wound area from adverse effects of oxidative agents [32]. CPPP possess a free radical-scavenging activity, which is a major naturally occurring antioxidant component of this compound [33]. CPPP possess anti-inflammatory effects [10], and it is implicated that the acceleration of wound healing produced by this compound might be due to its anti-inflammatory effects [34].

In this experiment, wound tissue homogenate from rats treated with CPPP showed significant antioxidant property via decreased MDA level and by elevated activity of CAT and SOD endogenous enzymes. Free radicals and ROS are continuously produced intracellularly by multiple sources, including the peroxisomes, mitochondria, and endoplasmic. These oxygen species directly cause cell injury. Therefore, wound tissues should be protected from oxidative stress through both extracellular and intracellular antioxidants [35]. The decrease in CAT and SOD antioxidant activities in wound tissue homogenates of the control rats may be attributed to the increased production of ROS which reduces the activity of these two enzymes [36]. The decrease in SOD and CAT enzymes activity in the wound tissue homogenate may result in a large number of deleterious effects. The topical application of CPPP on wound areas restores the activities of these two enzymes and may help to prevent the oxidative stress of the free radicals. Superoxide and hydroxyl radicals are essential mediators of oxidative stress and may be played significant roles in many clinical disorders. As noted above, reduced CAT and SOD enzymes activities were observed in wound tissue homogenates of the control rats, which might have resulted from the utilisation of these molecules for the decomposition of superoxide anions produced by lipid peroxidation. Decreased activities of these enzymes may produce a number of deleterious effects. CAT is thought to be an essential factor in several cellular functions and in the defence against many oxidative stresses. Dietary CAT suppresses oxidative stress *in vivo *[37]. Lipid peroxidation is a main pathophysiological result in various disorders [38].

TGF-*β* is an important peptide that controls the wound healing process, stimulates the attraction of cells to a wound area, and promotes the deposition of collagen fibers and intercellular ground substances [39]. TGF-*β* levels were remarkably lower in the CMC group than other groups and remarkably high in treated groups which is similar to the report of curcumin in enhanced TGF-*β* expression [40]. On the other hand, proinflammatory cytokines play a major role in wound healing process [41]; TNF-*α* was remarkably increased in the CMC-treated rats indicating remarkably a high inflammatory state in the wound tissue homogenate. Low and high dosage of CPPP application in the rat's wound improved the levels of TNF-*α*.

Masson's trichrome staining wound section revealed that there were remarkable improvements in collagen deposition and alignment following the application of CPPP to the wounds. In particular, the amount of collagen recovered was close to the standard drug level (Group 2). The capacity to stimulate collagen production in the wounded skin and to facilitate and accelerate the rate of wound healing closure without any irritation may provide a rationale for the preparation and development of a herbal “skin-repair.” Further experimental studies are required to assess the effects of CPPP on proliferation of fibroblasts *in vitro* which is one of the study limitations.

## 5. Conclusions

This study highlights the wound-healing activity of CPPP. Our results indicate that CPPP facilitates wound healing and increases fibroblast accumulation, collagen deposition, and vascularisation over CMC-treated rats. Furthermore, CPPP improves the activity of the endogenous antioxidant enzymes defence (CAT and SOD) and decreases the lipid peroxidation level (MDA) in the wound homogenate. Further studies are required to understand the real mechanism of wound healing process in CPPP. 

## Figures and Tables

**Table 1:** Effect of CPPP on percentage (%) of wound healing in experimental rats.

Groups	Day 1	Day 5		Day 10	
	Wound area (mm²)	Wound area (mm²)	Closure %	Wound area (mm²)	Closure %
CMC	320.0 ± 1.3	172.7 ± 0.8	46.1 ± 0.3	86.7 ± 1.3	72.9 ± 0.4
Intrasite gel	317.3 ± 0.8	96.7 ± 1.2*	69.5 ± 0.4*	19.3 ± 2.2*	93.9 ± 0.7*
CPPP LD	319.3 ± 1.9	150.7 ± 11.8*	52.8 ± 3.7*	26.0 ± 2.3*	91.9 ± 0.7*
CPPP HD	318.0 ± 3.1	134.7 ± 4.6*	57.7 ± 1.4*	24.0 ± 4*	92.5 ± 1.3*

Statistical analysis of the data was carried out using one way analysis of variance (ANOVA) and Tukey's post hoc test for average comparison on SPSS 18.0. Mean values ± SEM were used. Significance was defined as *P < 0.05 compared to CMC group.

**Table 2:** Effects of CPPP on SOD and CAT in tissue homogenates of dermal wounds in rats.

Type of dressings (twice daily) (0.2 mL/animal)	SOD (U/mg protein)	CAT (nmol/min/mL)
CMC	2.94 ± 0.09	22.89 ± 0.90
Intrasite gel	5.64 ± 0.33*	37.09 ± 1.05*
CPPP LD	3.35 ± 0.21	25.43 ± 0.65
CPPP HD	4.07 ± 0.12*	29.94 ± 0.90*

Statistical analysis of the data was carried out using one way analysis of variance (ANOVA) and Tukey's post hoc test for average comparison on SPSS 18.0. Mean values ± SEM were used. Significance was defined as *P < 0.05 compared to CMC group.

**Table 3:** Effects of CPPP on cytokines in tissue homogenates of dermal wounds in rats.

Type of dressings (twice daily) (0.2 mL/animal)	TGF-*β* (pg/mL)	TNF-*α* (pg/mL)
CMC	18.49 ± 0.34	84.31 ± 0.67
Intrasite gel	47.22 ± 0.75*	52.06 ± 0.71*
CPPP LD	25.30 ± 0.23*	45.95 ± 1.31
CPPP HD	46.22 ± 0.95*	55.94 ± 1.16*

Statistical analysis of the data was carried out using one way analysis of variance (ANOVA) and Tukey's post hoc test for average comparison on SPSS 18.0. Mean values ± SEM were used. Significance was defined as *P < 0.05 compared to CMC group.
